# The Relationship Between Sleep and Cognition in Children Referred for Neuropsychological Evaluation: A Latent Modeling Approach

**DOI:** 10.3390/children5030033

**Published:** 2018-02-28

**Authors:** Adrian Svingos, Sarah Greif, Brittany Bailey, Shelley Heaton

**Affiliations:** Department of Clinical & Health Psychology, University of Florida, Gainesville, FL 32610, USA; sgreif1021@ufl.edu (S.G.); bbailey92@phhp.ufl.edu (B.B.); sheaton@phhp.ufl.edu (S.H.)

**Keywords:** clinical, pediatric, cognitive, neuropsychology, sleep, structural equation modeling

## Abstract

Children with conditions affecting cognitive processes experience high levels of sleep disturbance, which may further compound the cognitive ramifications of their disorders. Despite this, existing studies in this area have been primarily confined to only particular diagnostic groups and/or a limited scope of sleep and cognitive parameters. The current study characterized the nature of sleep problems and examined the relationship between a wide range of sleep-related problems and cognitive functioning in a large (*N* = 103) diagnostically heterogeneous sample of youth (aged 6–16) referred for neuropsychological assessment. Structural equation modeling was used to examine the relationship between sleep-related problems (i.e., daytime sleepiness, sleep onset latency, sleep fragmentation, sleep time variability, sleep debt) and cognitive performance (i.e., executive functioning, sustained attention, memory, processing speed). Sleep fragmentation emerged as the most prominent sleep-related problem in the present sample. Structural equation modeling demonstrated a negative association between sleep-related problems and cognition that did not reach statistical significance (β = −0.084, *p* = 0.629). The current statistical approach may be used as a conceptual framework for future work examining these multi-dimensional constructs in a parsimonious fashion.

## 1. Introduction

Sleep has been described as one of the most important behavioral processes supporting healthy brain development and function [1,2]. Over a century of research in the area of sleep and cognition has unveiled a variety of neurocognitive functions of the mature brain that are susceptible to the negative effects of inadequate sleep [3,4]. Sleep may play an even more important role in the developing brain, as this is a critical time for new learning and skill acquisition [5]. Children with neuropsychological conditions (i.e., conditions affecting cognitive processes) experience higher rates of sleep disturbance than their healthy counterparts [6,7,8,9]. Common sleep problems among children with neuropsychological conditions include difficulties with daytime sleepiness [10,11,12], prolonged sleep onset latency [12,13,14], sleep fragmentation [14,15,16,17,18,19], sleep schedule variability [20,21], and sleep debt [12,13,14,22].

In healthy children, sleep disturbance has been associated with decreased cognitive performance across tasks of learning and memory [23], executive functioning [24,25,26], sustained attention [23,27], and processing speed [28,29,30,31]. Given that children referred for neuropsychological evaluation are more likely to experience cognitive deficits at baseline than healthy children, the impact of sleep disturbance is likely to further compound the cognitive ramifications of their disorders [32]. Despite this, a paucity of research has examined the relationship between a full range of sleep parameters and cognitive constructs in a heterogeneous clinical sample of patients referred for neuropsychological evaluation. Studies to date have primarily focused on target clinical populations (e.g., traumatic brain injury, attention-deficit/hyperactivity disorder), or have used heterogeneous clinical samples to examine the relationship between sleep and only one aspect of cognition (e.g., working memory) [33].

The present study utilized structural equation modeling (SEM) to comprehensively examine the relationship between sleep disturbance and neurocognitive dysfunction among children referred for neuropsychological evaluations. Structural equation modeling afforded the opportunity to parsimoniously examine the relationship between a variety of sleep-related problems and cognitive constructs, while accounting for their shared variance. It was thought that any observed associations between sleep and cognition that were maintained across a multitude of childhood neuropsychological conditions might point to critical areas for intervention. We were interested in first identifying which aspects of sleep disturbance were the most commonly observed within this diagnostically diverse sample and second, examining the broad association between sleep problems and cognition. It was hypothesized that children referred for neuropsychological testing would exhibit high levels of sleep disturbance characterized by difficulties with sleep onset and maintenance, daytime sleepiness, sleep quantity, and sleep time variability. It was also hypothesized that a negative association would be detected between sleep problems and neuropsychological performance such that increases in sleep problems would reflect greater levels of cognitive dysfunction.

## 2. Materials and Methods

Study participants were children referred for neuropsychological evaluation at a large southeastern university teaching hospital psychology clinic in the United States of America. Children were included in the study if (a) their parents provided consent for their clinical information to be stored in a research databank (approved by the University of Florida Institutional Review Board on 9/17/2015: IRB#201500639); (b) they were between the ages of 6 and 16; and (c) if they completed all study measures of interest (part of a core clinical assessment battery). Clinical data of interest were de-identified and extracted from the research databank using a third-party gatekeeper in accordance with a databank withdrawal protocol (approved by the University of Florida Institutional Review Board on 14 August 2015: IRB#201500554). One hundred and three cases (*N* = 103) were identified that completed all measures of interest and were included in statistical analyses. Participant ages ranged from 6 to 16 years with an average age of 10.62 (SD = 2.64) years. The sample was predominately male (62%), right-handed (88%), and White (77%). The average Full Scale IQ standard score of the sample was 91 (SD = 13.76), with IQs ranging from 53 to 129. Primary diagnosis of developmental and learning disorders (e.g., ADHD, specific learning disorder), neurological/other medical conditions affecting the brain (e.g., TBI, epilepsy, oncology), and mood or other psychiatric disorders represented approximately 68%, 26%, and 6% of the sample, respectively. Most children did not take attention medication on the day of their neuropsychological assessment (67%). Hollingshead scores of SES ranged from 11 to 77 with an average Hollingshead score of 35.93 (SD = 18.17). The majority of caregivers completing the sleep measure were identified as mothers (67.3%). Detailed participant demographic information is presented in Table 1.

Parent(s) or caregiver(s) accompanied each participant on the day of neuropsychological assessment and completed a demographic questionnaire that included the child’s age at time of assessment, gender, race, ethnicity, handedness, parental occupation, and highest level of parental education completed. Hollingshead scores (formulaic approximations of socioeconomic status based on parental occupation and highest level of education completed) were used to estimate child socioeconomic status (SES) [34]. Hollingshead scores were calculated for each adult caregiver within the child’s primary household according to published guidelines; when the child’s primary household was comprised of more than one adult caregiver, data from the highest SES approximation (i.e., lowest Hollingshead score) were used. Other relevant patient information (e.g., clinical diagnoses, Wechsler FSIQ, and medication status) was also extracted from the IRB-approved clinical databank. Though most of the patients received multiple diagnoses, a “primary diagnosis” was assigned under supervision of the licensed assessing clinician that reflected the most impairing neuropsychological condition that patients presented with at the point of evaluation.

All cognitive domains of interest were measured using published neuropsychological tests with strong psychometric properties [35,36,37]. Raw scores on cognitive tests were transformed into demographically-corrected, norm-referenced standardized scores in accordance with published test manuals. Learning and memory abilities were assessed using the General Memory Index (GMI) from the Children’s Memory Scale [35]. Participant index scores on the GMI were used to assess global memory processes (that include the child’s ability to initially learn or encode both visually and verbally presented stimuli).

Sustained attention capacity was measured using the Score! subtest of the Test of Everyday Attention for Children (TEA-Ch), a neuropsychological assessment tool used to assess different aspects of attentional and executive functioning in children ages 6 to 16 years old. Like the CMS, the TEA-Ch demonstrates strong psychometric properties and is used for multi-dimensional assessment of attention in pediatric neuropsychology clinics [36]. The Score! subtest requires children to accurately count audiotaped tones that are presented over the course of approximately 15 min.

Executive functioning was measured using the TEA-Ch Creature Counting subtest which loads strongly as a measure of broad executive functioning and was thus selected as the primary frontal-executive measure in the current study [38]. The TEA-Ch Creature Counting subtest requires children to accurately and effectively shift set by changing the direction of their counting (forward versus backward counting) when prompted with visual cues (“up” versus “down” arrows).

Processing speed was assessed using the Processing Speed Index (PSI) score derived from the Wechsler Intelligence Scale for Children-Fourth Edition (WISC-IV) [37]. The PSI score has strong psychometric properties and is derived from age-corrected norm-referenced performance on the two component subtests: Digit-Symbol Coding and Symbol Search [37]. The Digit-Symbol Coding subtest requires children to rapidly decode numbers into symbols based on a presented key. The Symbol Search subtest requires children to quickly decide if either of two symbols presented are repeated in a line of symbols presented to the right of the target set, and to mark these symbols accordingly.

Sleep parameters of interest were measured using the Heaton Children’s Sleep Questionnaire (HCSQ). The HCSQ is a 90-item caregiver-report sleep measure that was assembled for clinical use. The caregiver report was used as opposed to objective measures (e.g., actigraphy, polysomnography) because (1) the authors were interested in examining sleep-related problems that were clinically salient enough (i.e., disruptive enough) to be reported by parents, and (2) objective measures are typically not available within the context of an outpatient neuropsychology clinic. The HCSQ is comprised of sleep items identified from existing published sleep measures that have been validated for use in pediatric populations [39,40,41,42,43,44,45,46,47]. All HCSQ items utilize a five-point Likert rating scale ranging from Never (0) to Very often (4), which requires caregivers to rate the frequency of various sleep behaviors occurring across a “typical week during the past month.” The Likert scale is anchored to the frequency of occurrence in the target week as outlined in Table 2. Individual items from the HCSQ were used in order to calculate composite scores related to daytime sleepiness, sleep onset latency, sleep fragmentation, sleep time variability, and sleep debt. Items that were not worded in a problem-focused direction were reverse coded such that higher Likert values reflected increased sleep disturbance. Specific items used to calculate sleep-related problems composite scores are presented in Table 3.

Descriptive statistical analyses were completed using IBM-SPSS Statistics 22. Confirmatory factor analysis (CFA) and SEM were conducted within IBM AMOS 22. In order to assess the complex relationship between sleep and cognition, two latent factors were created that included all measures of sleep and cognitive functioning. These latent factors were first examined independently in CFAs and then included in a larger CFA and structural equation model which assessed the extent to which sleep problems were associated with cognitive dysfunction among children with neuropsychological conditions. Multivariate normality was assessed using Mardia’s coefficient, a widely used estimate of multivariate normality. Composite scores from Likert scale questionnaires are particularly likely to appear non-normal because of differences in shape of distribution from those typically used in parametric testing [48,49]. Bootstrapping (1000 samples) was employed for all CFA and SEM analyses with significant threats to normality that could not be explained by the shape of the distribution.

Squared multiple correlations were used to determine the percent of variance within each measure explained by the particular factor. Model fit was assessed using a variety of widely used goodness-of-fit indices [50,51,52]. Fit index descriptions and corresponding thresholds for appropriate model fit are presented in Table 4.

Modification indices generated were considered one at a time and incorporated only if theoretically sound [57]. A threshold of 3.84 for significant chi-square improvement was used for detection of modification indices. Nested model fit chi-square tests were conducted to compare any modified models to the null or previous model. If adjustments suggested by modification indices significantly improved model fit, they were included in the subsequent model.

## 3. Results

### 3.1. Descriptive Statistics

Descriptive statistics for all sleep and cognitive variables included in statistical analyses are presented in Table 5.

### 3.2. Sleep-Related Problems

At the item-level, sleep fragmentation (i.e., your child awakens once during the night), daytime sleepiness (i.e., your child has a problem with sleepiness during the day), sleep onset latency (i.e., your child has difficulty falling asleep), and sleep debt (i.e., your child sleeps too little) were the most frequently observed, with 34.7%, 22.1%, 22.1%, and 21.1% of ratings in the “Often”–“Very often” range for each item, respectively. Of the sleep-related problems composites, sleep fragmentation emerged as the composite with the highest average frequency of ratings in the “Often”–“Very often” range (21.1%). The sleep onset latency composite featured the second highest average frequency of ratings in the “Often”–“Very often” range (19.5%), followed by sleep debt (15.3%), daytime sleepiness (12.6%), and sleep time variability (11.6%).

### 3.3. Confirmatory Factor Analysis: Sleep-Related Problems

The overall model fit of the CFA for sleep-related problems was poor, χ^2^ (5) = 18.22, *p* < 0.05. Additional fit indices are displayed in Table 6 (Model 1). Unstandardized estimates, standardized regression weights (loadings), significance of loadings, and squared multiple correlations are presented in Table 7 (Model 1). The daytime sleepiness unstandardized loading was fixed to “1” for all analyses and thus the significance of its loading on the latent factor could not be determined. All other sleep composites loaded significantly (*p* = 0.001) with respect to the sleep-related problems factor. 

Standardized regressions ranged from 0.380 to 0.833. The strongest loading was sleep debt, followed by sleep onset latency, sleep fragmentation, sleep time variability, and daytime sleepiness. The sleep-related problems factor explained over half of the observed variance in sleep debt (69.4%) and sleep onset latency (51.5%). Marginal proportions of observed variance in sleep fragmentation (35.7%) and sleep time variability (30.2%) were explained by the sleep-related problems factor. The sleep-related problems factor explained a relatively small portion of variance in daytime sleepiness (14.4%).

Modification indices were available and considered for theoretically sound inclusion in the subsequent model. The modification index with the highest predicted discrepancy decrease (MI = 8.171) suggested that daytime sleepiness and sleep fragmentation error variances should be permitted to freely correlate. Given that sleep fragmentation may undoubtedly contribute to experience of daytime sleepiness, these measures likely share error variance and were allowed to correlate in the proceeding model, described below.

### 3.4. Confirmatory Factor Analysis: Modified Sleep-Related Problems

**Figure 1:**
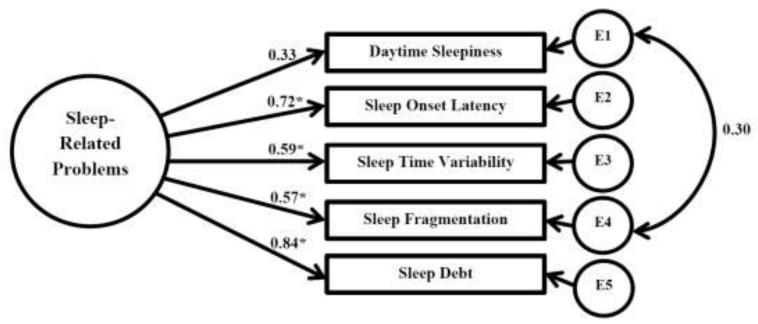
Confirmatory factor analysis: Modified sleep-related problems; * Significant (*p* ≤ 0.05); E—error.

The changed model with freely correlating daytime sleepiness and sleep fragmentation error variances displayed improvement across all goodness-of-fit indices as well as statistically significant improvement in model fit, Δχ^2^ = 8.66, Δ df = 1, *p* = 0.003. Therefore, this improved model was used in in the two-factor CFA with sleep-related problems and cognition. All fit indices are displayed in Table 6 (Model 2). Of note, sleep measures remained significantly loaded on the sleep-related problems factor (*p* < 0.05). Standardized loadings were comparable to the previous model. The relationship between sleep fragmentation and daytime sleepiness was positive and significant, r = 0.304, *p* = 0.007, such that greater sleep fragmentation was associated with greater daytime sleepiness. A visual representation of the sleep-related problems confirmatory factor analysis is presented in Figure 1.

### 3.5. Confirmatory Factor Analysis: Cognition

**Figure 2:**
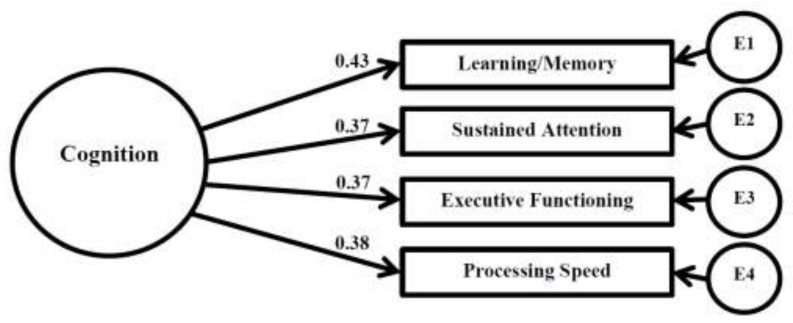
Confirmatory Factor Analysis: Cognition; * Significant (*p* ≤ 0.05); E = error.

The CFA model with latent cognition factor demonstrated excellent fit, χ^2^ (2) = 0.702, *p* = 0.704. Additional fit indices are displayed in Table 6 (Model 3). Unstandardized estimates, standardized regression weights (loadings), significance of loadings, and squared multiple correlations are presented in Table 7 (Model 3). None of the cognitive parameters significantly loaded on the latent cognition construct, although these measures appeared to share a rather uniform portion of variance explained in the cognition factor (14.5–18.4%). A visual representation of the cognition confirmatory factor analysis is presented in Figure 2.

### 3.6. Confirmatory Factor Analysis: Sleep-Related Problems and Cognition

The two-factor CFA model including sleep-related problems and cognition displayed relatively good fit according to several fit indices (i.e., CFI, GFI, (χ^2^)/df, RMSEA,) although the chi-square suggests a significant likelihood for discrepancy in fit, χ^2^ (25) = 38.47, *p* < 0.042. All fit indices are displayed in Table 6 (Model 4). Sleep onset latency, sleep time variability, sleep fragmentation, and sleep debt all significantly loaded on the sleep-related problems factor, *p* ≤ 0.05. All other loadings failed to reach statistical significance. Factor inter-correlation statistics indicated that the sleep-related problems factor and cognition factor were negatively associated, although their association was small and failed to reach statistical significance, r = −0.084, *p* = 0.630. Unstandardized estimates, standardized regression weights (loadings), significance of loadings, and squared multiple correlations are presented in Table 7.

### 3.7. Structural Equation Model: Sleep-Related Problems and Cognition

**Figure 3:**
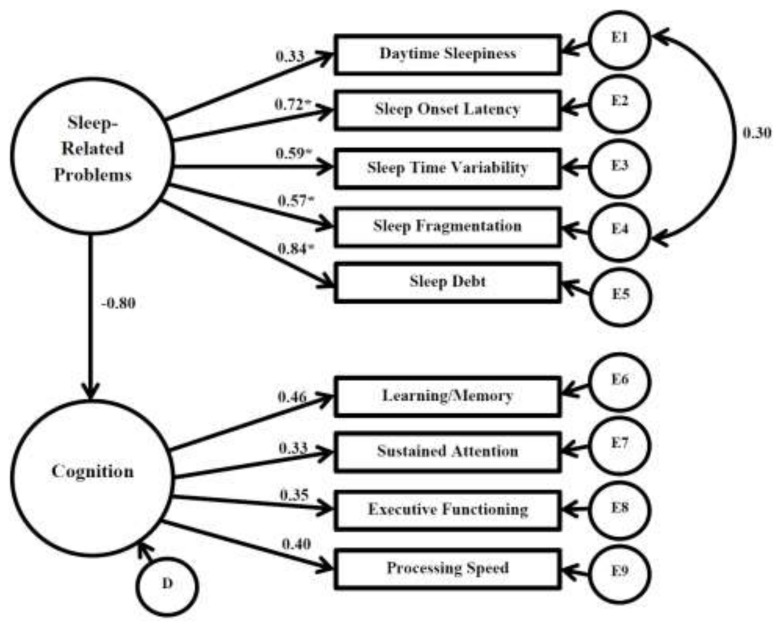
Structural Equation Model: Sleep-Related Problems and Cognition; * Significant (*p* ≤ 0.05); E—error; D—disturbance.

Given that factor structure remained unchanged from Model 4, all fit indices remained identical. All unstandardized estimates, standardized regression weights (loadings), significance of loadings, and squared multiple correlations remained consistent with previous models and are presented in Table 7. The SEM allowed for inferential assessment of causation between latent sleep-related problems and cognition. The standardized regression weight from the sleep-related problems to cognition factor was negative, but insignificant (β = −0.084, *p* = 0.629). A visual representation of the sleep-related problems and cognition SEM is presented in Figure 3.

## 4. Discussion

The current study sought to capture the full breadth of sleep-related problems commonly observed in various pediatric diagnostic groups and to parsimoniously assess the extent to which more broad associations may exist between these sleep-related problems and cognition in a heterogeneous clinical sample. Here, we discuss key study results and implications, relevant threats to interpretability of findings, as well as future directions and rationale for replication and improvement of presently employed methodology.

Among the sleep-related problems composites, sleep fragmentation emerged as the composite with the highest average frequency of ratings in the “Often”–“Very often” range (21.1%). As such, sleep fragmentation may be one of the most commonly observed and/or most prominent sleep-related problems for children with disorders affecting the central nervous system. When considered in the context of existing research, findings illustrate the extent to which children referred for neuropsychological assessment may be more likely to exhibit sleep-related problems than their healthy peers. In our clinical pediatric sample, the prevalence of reported nighttime awakenings was over twice that which has been demonstrated among healthy children within a similar age range [58]. This was also the case for reported difficulty with sleep onset (i.e., your child has difficulty falling asleep).

In our heterogeneous clinical sample, 21.1% of caregivers endorsed sleep onset difficulties occurring more than once per week, compared to only 8.9% of caregivers of healthy children within a similar age range [58]. Interestingly, the opposite pattern was observed for sleep-time variability and specifically bed time variation, which was far less prevalent in our sample (12.6%) than among a group of similarly aged healthy children (38.6%) [58].

Although the HCSQ has not yet been experimentally validated, sleep composites displayed significant and strong factor loadings on the sleep-related problems factor across all models, suggesting that these composites were rather stable in their association with the underlying sleep-related problems factor. The sleep composites also had large proportions of unique variance that were explained by the latent sleep-related problems factor, suggesting that these factors may reflect distinguishable, but largely associated constructs within the sleep-related problems factor. Of note, over 70% of the observed variance in sleep debt was explained by the sleep-related problems factor in the structural equation model. As mentioned in the introduction of this paper, sleep fragmentation and delayed sleep onset may contribute to one’s subjective experience that they are not getting enough sleep. Study results corroborated with the a priori expectation that sleep debt would emerge as an important parameter to consider in association with cognition. Results also indicate that parental report of sleep debt may serve as a useful indicator of more global sleep-related problems.

Although sleep parameters displayed strong loadings on their latent factor, the overall sleep-related problems model fit was relatively poor, even after modification. This may have been due to asymmetry of residual covariances. Slight asymmetries of residual covariance can signal poor fit, even in a model that correctly estimates most of the residual covariances for its data [59]. Observed residual covariance asymmetry within the sleep- related problems CFA warrants further investigation of the potential moderators of asymmetry within this model. Multi-group modeling to assess the modifying effects of diagnostic group and potential factor structural invariance would be particularly interesting. Do sleep problems load similarly on the sleep-related problems factor across different diagnostic groups? Incorporating objective measurement of sleep parameters (e.g., multiple sleep latency tests for daytime sleepiness, wrist actigraphy for sleep onset latency and wake after sleep onset) may prove helpful in providing additional construct stability prior to assessing multi-group structural invariance or other potential factors underlying the observed covariance asymmetry within the current model.

Study results also indicate that structural equation modeling may be appropriate for assessment of complex associations (i.e., the relationship between sleep-related problems and cognitive functioning) in heterogeneous clinical pediatric populations. Specifically, the cognition CFA displayed excellent model fit, aligning with existing literature suggesting that cognitive outcomes are often most appropriately conceptualized with multivariate design [60]. Despite this, cognitive measures in the present study did not exhibit large portions of variance explained by the latent cognition factor. This was rather surprising, as cognitive variables are typically strong candidates for multivariate study design in that they tend to share large portions of variance [61]. Cognitive measures may have contained large portions of variance explained by factors other than the latent cognition factor because of vast sample heterogeneity (i.e., central nervous system dysfunction with dramatically different etiologies).

The present study is limited by its inherently correlational design. Although structural equation modeling may be used to infer causation [49,51], causal relationships should undoubtedly be replicated in the context of a traditional longitudinal study design. Moreover, model specifications revealed several limitations to the overall interpretability of study findings. Weak factor loadings and insufficiently squared multiple correlations for measures within initial CFA model, for example, restrict the extent to which any between-factor associations (i.e., correlations, regression weights) can be interpreted. In addition, the HCSQ items used to derive sleep-related problems composites do not provide insight to the etiological underpinnings of specific sleep concerns. This in turn may hinder the extent to which findings may be used to guide selection of appropriate interventions. Another limitation to the interpretability of findings exists in that poor model fit prompted inspection and incorporation of modification indices. Byrne (2001) suggests that inclusion of modification indices returns analyses to an exploratory realm, and that using modification indices warrants replication of findings in an entirely unique sample [52].

Despite the above-mentioned limitations, the final SEM displayed adequate fit according to multiple goodness-of-fit indices. While problems with construct validity of the model prevent conclusions regarding the relationship between sleep and cognitive factors in this population, results indicate that structural equation modeling may be an appropriate approach for future assessment of sleep-related problems and cognition in heterogeneous clinical pediatric populations. Future studies should examine IQ as a potential covariate in this model and should consider including multiple measures for cognitive outcomes of interest, especially given that some of the cognitive domains of interest were measured with scaled scores from only one stand-alone subtest as part of a neuropsychological assessment (i.e., executive functioning and sustained attention). Investigators interested in replicating study findings may first consider exploratory factor analytic approaches to create strong latent factors for later inclusion in a SEM. While theoretical backing for a confirmatory factor analytic approach was likely sufficient in the current study, improved model fit in CFAs should be obtained in future attempts to examine these complex constructs in a multivariate fashion. These adjustments will likely produce more reliable and meaningful findings.

Although the current study meets many of the suggested guidelines for sufficient sample size [62], more complex and interesting questions could be answered with a larger sample size. For example, future studies with larger sample size would be able to look at the moderating effects of age by testing parameter invariance with multi-group SEM modeling. This would be particularly interesting, given how little is known about the associations between sleep and cognition at different points in a child’s developmental course.

## 5. Conclusions

The current study provides a methodological framework for parsimoniously assessing the relationship between a variety of sleep and cognitive parameters in a heterogeneous pediatric sample. Latent multivariate modeling allows for careful analysis and incorporation of measurement error, as well as inherent control for multicollinearity of predictors and outcomes. Given that patients with neuropsychological disorders are more inclined to experience sleep problems than healthy children of the same age and that sleep problems may further compound the cognitive ramifications associated with these CNS disorders, it is imperative that researchers continue to develop appropriate models to assess these complex associations. Identification of global associations that exist between sleep and cognition in the context of a brain development and dysfunction may illuminate potential etiological pathways that could be targeted for future study, as well as clear targets for the role of behavioral sleep medicine in cognitive rehabilitation.

## Figures and Tables

**Table 1:** Sample Demographics.

Variable	Group	Frequency (%)
Primary Diagnosis *	ADHD	50.5
	Traumatic brain injury	6.8
	Cancer/brain tumor	7.8
	Specific learning disorder	8.7
	Epilepsy	6.8
	Other CNS disorder	4.9
	Mood disorder	1.9
	Other psychiatric	3.9
Race	White	76.7
	Black or African-American	16.5
	Multiracial	5.8
	Asian	1.0
Ethnicity	Hispanic or Latino	6.8
	Not Hispanic or Latino	93.2
Handedness	Right	88.3
	Left	8.7
	Ambidextrous	2.9

* Primary diagnosis represents the most impairing neuropsychological condition diagnosed; ADHD—attention-deficit/hyperactivity disorder; other CNS disorder—other medical condition affecting central nervous system functioning, mood disorder—anxiety or depressive disorders, other psychiatric—other psychiatric disorders of behavior or mood.

**Table 2:** Likert Scale Labels and Designated Frequency Anchors for HCSQ Items.

Score	Label	Frequency
0	Never	Never occurs during a typical week of month (i.e., does not happen)
1	Rarely	Occurs less than 1 day/night during a week, but at least once in a month
2	Sometimes	Occurs 1 day/night during a week, but at least once in a month
3	Often	Occurs 2–4 days/nights during a week
4	Very often	Occurs 5–7 days/nights during a week

**Table 3:** Sleep-Related Problems Composite Composition.

Composite	HCSQ Item
Daytime Sleepiness	*During the daytime, your child …*
	64. Has a problem with sleepiness during the day
	65. Seems tired
	66. Teachers or other supervisors comment that he/she appears sleepy
	67. Feels an irresistible urge to take a nap during the day
	68. When awake, disrupts family activities because of sleepiness
	69. Yawns a lot during the day
	70. Takes a nap during the day
	71. Falls asleep if sent to room for misbehaving
	72. Is very sleepy while watching TV
	73. Falls asleep while watching TV
	74. Is very sleepy while riding in a car
	75. Falls asleep while riding in a car
Sleep Onset Latency	*Your child …*
	7. Falls asleep within 20 min after going to bed *
	8. Has difficulty falling asleep
Sleep Fragmentation	*At night time, your child …*
	33. Awakes once during the night
	34. Awakes more than once during the night
	35. Awakens during the first 2 h after falling asleep
Sleep Time Variability	*Your child …*
	4. Sleeps about the same amount each day (combining nighttime and naps) *
	5. Has a regular bedtime routine *
	6. Goes to bed at the same time at night *
Sleep Debt	*Your child …*
	1. Sleeps too little
	2. Sleeps the right amount *

* Item was reverse-coded.

**Table 4:** Goodness-of-Fit Indices and Thresholds.

Index	Abbreviation	Threshold	Reference
Chi-square	χ^2^	>0.05	[51]
Normed Comparative Fit	CFI	≥0.90	[53]
Goodness-of-Fit Index	GFI	≥0.90	[54]
Adjusted-Goodness-of-Fit Index	AGFI	≥0.90	[54]
Chis-square/Degrees of Freedom	(χ^2^)/df	<2	[50,55]
Root Mean Square Error of Approximation	RMSEA	<0.05 or <0.08	[56]

**Table 5:** Descriptive Statistics for Sleep-Related Problems and Cognition.

Factor	Construct	Mean (SD)	Observed Range	Possible Range
Sleep-Related Problems ^a^	Daytime Sleepiness	12.7 (10.0)	0–48	0–48
	Sleep Onset Latency	2.7 (2.1)	0–8	0–8
	Sleep Fragmentation	4.2 (3.2)	0–12	0–12
	Sleep Time Variability	2.4 (2.7)	0–12	0–12
	Sleep Debt	2.32 (1.9)	0–7	0–8
Cognition	Learning/Memory ^b^	92.3 (18.4)	50–139	40–160
	Sustained Attention ^c^	7.9 (3.8)	1–14	1–19
	Executive Functioning ^c^	5.9 (4.0)	1–17	1–19
	Processing Speed ^b^	87.5 (13.0)	50–126	40–160

^a^ Sleep variable values represent the sum of Likert scale ratings (0–4) for the sleep items identified for the construct; ^b^ Standard Score (Mean = 100, SD = 15); ^c^ Scaled Score (Mean = 10, SD = 3).

**Table 6:** Fit Indices and Model Comparison.

Model	df	χ^2^	*p*	CFI	GFI	AGFI	(χ^2^)/df	RMSEA (*p*)	Δ df	Δ χ^2^	*p*
1	5	18	0.003	0.889	0.942	0.827	3.644	0.161 (0.01)			
2	4	9.6	0.049	0.958	0.967	0.877	2.389	0.117 (0.01)	1	8.7	0.003 *
3	2	0.7	0.704	1	0.997	0.983	0.351	0.000 (0.76)			
4	25	38	0.042	0.908	0.935	0.883	1.539	0.073 (0.20)			

Model 1—Null Sleep-Related Problems CFA; Model 2—Modified Sleep-Related Problems CFA; Model 3—Cognition CFA; Model 4—Sleep-Related Problems and Cognition CFA; * Significant model improvement from Model 1.

**Table 7:** Factor loadings and squared multiple correlations.

Model	Measure	Unstandardized Estimate	SE	*p*	β	Squared Multiple Correlation
1	Sleep Onset Latency	0.396	0.115	0.001	0.718	0.515
	Sleep Time Variability	0.426	0.132	0.001	0.589	0.346
	Sleep Fragmentation	0.504	0.155	0.001	0.596	0.355
	Sleep Debt	0.426	0.122	0.001	0.833	0.694
	Daytime Sleepiness	1.00	-	-	0.380	0.144
2	Sleep Onset Latency	0.459	0.155	0.003	0.723	0.522
	Sleep Time Variability	0.493	0.174	0.005	0.591	0.350
	Sleep Fragmentation	0.554	0.171	0.001	0.569	0.324
	Sleep Debt	0.498	0.166	0.003	0.844	0.713
	Daytime Sleepiness	1.00	-	-	0.330	0.109
3	Learning/Memory	1.00	-	-	0.429	0.184
	Sustained Attention	0.177	0.116	0.127	0.366	0.134
	Executive Functioning	0.188	0.123	0.127	0.368	0.135
	Processing Speed	0.629	0.409	0.124	0.381	0.145
4	Sustained Attention	0.147	0.099	0.138	0.327	0.107
	Memory	1.00	-	-	0.459	0.211
	Processing Speed	0.616	3.99	1.22	0.400	0.160
	Executive Functioning	0.167	0.110	0.129	0.350	0.122
	Daytime Sleepiness	1.00	-	-	0.330	0.109
	Sleep Onset Latency	0.460	0.154	0.003	0.724	0.524
	Sleep Time Variability	0.492	0.173	0.004	0.592	0.350
	Sleep Fragmentation	0.554	0.170	0.001	0.570	0.324
	Sleep Debt	0.496	0.165	0.003	0.842	0.709

Model 1—Null Sleep-Related Problems CFA; Model 2—Modified Sleep-Related Problems CFA; Model 3—Cognition CFA; Model 4—Sleep-Related Problems and Cognition CFA.
